# Risk Factors for Primary *Clostridium difficile* Infection; Results From the Observational Study of Risk Factors for *Clostridium difficile* Infection in Hospitalized Patients With Infective Diarrhea (ORCHID)

**DOI:** 10.3389/fpubh.2020.00293

**Published:** 2020-07-17

**Authors:** Kerrie Davies, Jody Lawrence, Claire Berry, Georgina Davis, Holly Yu, Bing Cai, Elisa Gonzalez, Ida Prantner, Andrea Kurcz, Ioana Macovei, Hanna Pituch, Elena Nováková, Otakar Nyč, Barbara Gärtner, Fabian K. Berger, Monica Oleastro, Oliver A. Cornely, Maria J. G. T. Vehreschild, Louise Pedneault, Mark Wilcox

**Affiliations:** ^1^Healthcare Associated Infections Research Group, Leeds Teaching Hospitals NHS Trust and University of Leeds, Leeds, United Kingdom; ^2^Vaccine Research and Development, Pfizer Inc., Pearl River, NY, United States; ^3^Department of Hospital Epidemiology and Hygiene, National Center for Epidemiology, Budapest, Hungary; ^4^Cantacuzino National Medico-Military Institute for Research and Development, Bucharest, Romania; ^5^Department of Medical Microbiology, Medical University of Warsaw, Warsaw, Poland; ^6^Department of Microbiology and Immunology in Jessenius Faculty of Medicine Martin, Comenius University Bratislava, Martin, Slovakia; ^7^Department of Medical Microbiology, Second Faculty of Medicine, Charles University in Prague and Motol University Hospital, Prague, Czechia; ^8^Germany National Reference Centre for Clostridiodies Clostridium difficile, Institute of Medical Microbiology and Hygiene, Saarland University of Medical Center, Homburg, Germany; ^9^German National Reference Center for Clostridioides (Clostridium) Difficile, Institute of Medical Microbiology and Hygiene, Saarland University, Saarbrücken, Germany; ^10^Department of Infectious Diseases, National Institute of Health Dr. Ricardo Jorge, Lisbon, Portugal; ^11^Cologne Excellence Cluster on Cellular Stress Responses in Aging-Associated Diseases (CECAD), Department I of Internal Medicine, Clinical Trials Center Cologne (ZKS Köln), University of Cologne, Cologne, Germany; ^12^Department I of Internal Medicine, Germany and German Centre for Infection Research (DZIF), University Hospital of Cologne, Partner Site Bonn-Cologne, Cologne, Germany

**Keywords:** *Clostridium difficile*, risk factor, case control study, antibiotics, in-patients

## Abstract

**Background:** There are inconsistent data on the risk factors for *Clostridium difficile* infection (CDI) in the literature.

**Aims:** To use two *C. difficile* infection (CDI) case-control study groups to compare risk factors in hospitalized patients with diarrhea across different countries.

**Methods:** A multi-center group of CDI cases/controls were identified by standardized testing from seven countries from the prior EUropean, multi-center, prospective bi-annual point prevalence study of *CLostridium difficile* Infection in hospitalized patients with Diarrhea (EUCLID). A second group of CDI cases/controls was identified from a single center in Germany [parallel study site (PSS)]. Data were extracted from the medical notes to assess CDI risk factors. Univariate analyses and multivariate logistic regression models were used to identify and compare risk factors between the two groups.

**Results:** There were 253 and 158 cases and 921 and 584 controls in the PSS and EUCLID groups, respectively. Significant variables from univariate analyses in both groups were age ≥65, number of antibiotics (OR 1.2 for each additional antibiotic) and prior hospital admission (all *p* < 0.001). Congestive heart failure, diabetes, admission from assisted living or Emergency Department, proton pump inhibitors, and chronic renal disease were significant in PSS (all *p* < 0.05) but not EUCLID. Dementia and admitted with other bacterial diseases were significant in EUCLID (*p* < 0.05) but not PSS. Following multivariate analyses, age ≥ 65, number of antibiotics and prior hospital admission were consistently identified as CDI risk factors in each individual group and combined datasets.

**Conclusion:** Our results show that the same CDI risk factors were identified across datasets. These were age ≥ 65 years, antibiotic use and prior hospital admission. Importantly, the odds of developing CDI increases with each extra antibiotic prescribed.

## Introduction

The importance of *Clostridium difficile* as a healthcare-associated infection is well-established. In addition, the US Centers for Disease Control and Prevention (CDC) has designated this pathogen as one of the top three antibiotic resistant threats, emphasizing its impact on both patients and the healthcare economy (1–4).

New treatment options are being developed (5–8), but prevention of *C. difficile* infection (CDI) is a key goal. Optimal infection control practices, including antimicrobial stewardship, can significantly reduce CDI incidence and transmission (9). Vaccination against *C. difficile* and interventions to block the deleterious effects of antibiotics on the gut microbiome are being pursued as prevention options (6). Notably, however, preventative approaches require appropriate identification of patients at risk to optimize both the feasibility of clinical trials and ultimately the cost-effectiveness of such interventions.

Some CDI risk factors have been consistently reported, such as advancing age, hospital exposure/contact, and exposure to antibiotics (10). Damage to the host gut microbiome by antibiotics, and indeed certain other drugs can lead to proliferation of *C. difficile* and disease (11). Although almost all classes of antibiotics have been implicated in increasing the risk of CDI some, such as fluoroquinolones, may be key drivers of disease (9, 10). In the case of fluoroquinolones, which cause relatively little damage to the gut microbiome, the proliferation of a fluoroquinolone resistant strain resulted in outbreaks of CDI worldwide (12). These outbreaks decreased in the UK after the strict restriction of the use of fluoroquinolones (9). Prior hospitalization has been demonstrated as a risk factor for CDI even after adjusting for increasing age (13), with longer duration of stay associated with higher risk (14). It should be noted that not only is age a risk factor for CDI, deaths are also disproportionally higher in the elderly population, with 84% of deaths due to CDI in those >65 years old in 2011 (2).

In contrast some studies examining CDI risk factors have revealed conflicting information (15–18). For example, the role of proton pump inhibitors (PPIs) as a risk factor for CDI is controversial, possibly due to ascertainment bias relating to choice of control patients (19). Risk prediction models have had variable success; models developed in single center derivation cohorts have not proved robust in validation cohorts (20, 21), possibly due to the selection of the derivation cohort. Furthermore, interpretation of some CDI risk factors studies can be complicated by the following: (1) inclusion of cases with recurrent infection, rather than only those with primary disease, (2) use of non-robust case definitions, and (3) use of different diagnostic methodologies (16, 22–24). Our study aimed to determine the risk factors for developing primary CDI in hospitalized patients and compare the risks across distinct patient groups.

## Materials and Methods

### Case/Control Study

We used two groups of CDI cases and controls, defined according to standardized laboratory testing, (22–24) across multiple hospitals and countries in Europe. The first group of cases and controls were based on a point prevalence study of CDI [EUropean, multi-center, prospective bi-annual point prevalence study of *CLostridium difficile* Infection in hospitalized patients with Diarrhea (EUCLID)] and included 59 hospitals in seven countries across Europe that were identified as having higher rates of infection (Czech Republic, Germany, Hungary, Poland, Portugal, Romania, and Slovakia) (22). The EUCLID CDI cases and controls were collected during 2 separate days, one in winter 2012/2013 and the other in summer 2013. The second group of CDI cases and controls [referred to as parallel study site (PSS)] was chosen as this center utilized the same standardized CDI testing methodology (23, 24) as that used in the EUCLID study; testing was conducted between July and October 2015 at the University Hospital Cologne, Germany, a specialist cancer center.

From these predefined patient groups, we conducted a retrospective case-control study to determine the risk factors for CDI in hospitalized patients. For inclusion in the study, a case was defined as a patient with a positive result for both glutamate dehydrogenase (GDH) and *C. difficile* toxins from their diarrheal stool sample within the study period (PSS) or on the day of sampling (EUCLID). A control was defined as a patient who had a stool sample taken and tested in the time period of interest at the study site which was negative for CDI (GDH negative/toxin negative). For each confirmed CDI case, four geographically matched controls (i.e., patients with diarrhea that was shown by standardized testing not to be due to CDI) were randomly selected from each respective hospital. Risk factor data were extracted from available medical records via a standardized clinical report form (CRF), and were sent to the study coordinator for upload into the central database.

### Analysis

The index sample was the sample that defined entry into the study [either positive (cases) or negative (controls)]. For the analysis population, participants were removed from control groups if they had evidence of previous CDI within the preceding 8 weeks or within the subsequent 8 weeks of the index sample. Cases were classified as primary CDI where there was no positive toxin text in the 8 weeks prior to the index sample; otherwise cases were classified as recurrence. In addition, for the multivariate analysis all cases that had a previous toxin positive fecal sample were removed, so ensuring that only primary (and not recurrent) CDI cases were included for risk factor analyses; matched controls were not removed for this analysis.

Distribution of demographics, concomitant pathogens, medical conditions, medical procedures, and medications were tabulated by cases and controls. Where data were continuous variables, means, and standard deviations were calculated; where these data had a highly skewed distribution, medians, 25th and 75th percentiles were also calculated. Where data were categorical variables, the number, and percentage of participants within each category were calculated. The percentage of missing values was reported for each variable.

Univariate analysis was first used to assess risk factors; continuous variables were compared between cases and controls by *t*-test; where skewed, variables were also compared by Mann–Whitney test; categorical variables were compared by chi-squared (Fishers exact where numbers were below 5). Unadjusted odds ratios were calculated for association of CDI with identified variables of interest. For the multivariate analysis, unconditional logistic regression analysis was performed using stepwise forward selection with case/control as dependent variables, including identified variables of interest from the univariate analysis and a cut-off of *p* = 0.2. The same multivariate analysis approach was performed for each group (PSS, EUCLID) and for the entire data set (both groups combined).

### Ethics

Ethical approval for this study was gained from the University of Leeds (SoMREC/14/085) and in those participating countries where it was required (Germany, Hungary, Poland, Romania, and Slovakia). Ethical approval in Germany was sought on a regional (state) basis, in response to different regional requirements.

## Results

### Patient Population

There were 253 and 158 cases and 921 and 584 controls in the PSS and EUCLID groups, respectively, after removing controls with evidence of prior or subsequent CDI within 8 weeks (as described in the Methods section). There were slightly more males than females, both overall (ratio 1.26 males:1 females) and for each group (PSS ratio 1.26, EUCLID 1.25) (Table 1). The median age of all patients in the PSS group was significantly older than in EUCLID patients (67 vs. 60 years, *p* < 0.001); however, the median ages of cases within each group were similar (PSS cases median age 71 vs. 72 years for EUCLID cases; *p* = 0.885).

**Table 1:** Age and gender for all participants, per group, and per cases and controls.

	**PSS**			**EUCLID**		
	**Cases (%)**	**Controls (%)**	**Total number of participants**	**Cases (%)**	**Controls (%)**	**Total number of participants**
Total	253 (100)	921 (100)	1174	158 (100)	584 (100)	742
**Age category**						
<18 years	0 (0)	13 (1.4)	13	16 (10.1)	125 (21.4)	141
18–49 years	43 (17.0)	203 (22.0)	246	17 (10.8)	110 (18.8)	127
50–64 years	44 (17.4)	237 (25.7)	281	27 (17.1)	121 (20.7)	148
65–84 years	135 (53.4)	414 (45.0)	549	77 (48.7)	189 (32.4)	266
All ≥50 years	210 (83.0)	705 (76.5)	915	125 (79.1)	349 (59.8)	474
All ≥65 years	166 (65.6)	468 (50.8)	634	98 (62.0)	228 (39.0)	326
All ≥85 years	31 (12.3)	54 (5.9)	85	21 (13.3)	39 (6.7)	60
**Gender**						
Female	105 (41.5)	415 (45.1)	520	74 (46.8)	253 (43.3)	327
Male	148 (58.5)	506 (54.9)	654	83 (52.9)	327 (56.0)	410

### Univariate Analyses

Cases were significantly older than controls in both groups. In the PSS and EUCLID groups, respectively, the median age of cases was 71 and 72 years while the median age of controls was 65 and 58 years (*p* < 0.001 for both groups). Age ≥ 65 years was significantly associated with cases of CDI compared with controls (both *p* < 0.001, Table 2).

**Table 2:** Categorical variable with a statistically significant difference between cases and controls within at least one of the participant cohorts (risk factors consistently significant in both cohorts are shown in bold).

	**PSS**				**EUCLID**			
**Variables**	**Cases [*n* (%)]**	**Controls [*n* (%)]**	***X*^**2**^*P*-value**	**Odds ratio (95% CI)**	**Cases [*n* (%)]**	**Controls [*n* (%)]**	***X*^**2**^*P*-value**	**Odds ratio (95% CI)**
**Age** **≥** **65**	**166** ** (65.6)**	**468** ** (50.8)**	**<0.001**	**1.84** ** (1.4–2.5)**	**80** ** (63.0)**	**156** ** (37.2)**	**<0.001**	**2.40** ** (1.6–3.5)**
**Medical specialty where the participant was located at the time of the index sample**								
Infectious Diseases	1 (0.4%)	32 (3.0%)	0.005	0.110 (0.0–0.8)	20 (12.8%)	107 (18.5%)	0.095	0.646 (0.4–1.1)
Oncology	53 (21.0%)	254 (27.6%)	0.034	0.696 (0.5–1.0)	4 (2.6%)	24 (4.2%)	0.481	0.602 (0.2–1.8)
Renal Medicine/Nephrology	39 (15.4%)	85 (9.2%)	0.005	1.792 (1.2–2.7)	12 (7.7%)	29 (5.0%)	0.204	1.567 (0.8–3.2)
General Surgery	7 (2.8%)	7 (0.8%)	0.017	3.715 (1.3–10.7)	10 (6.4%)	25 (4.3%)	0.287	1.504 (0.7–3.2)
Gastroenterology	5 (2.0%)	6 (0.7%)	0.240	ns	12 (7.7%)	89 (15.4%)	0.012	0.451 (0.2–0.8)
**Reason for admission**								
Malignant neoplasms	34 (13.5%)	185 (20.1%)	0.017	0.619 (0.4–0.9)	n/a	n/a	n/a	n/a
Renal failure	21 (8.3%)	41 (4.5%)	0.015	1.947 (1.1–3.4)	5 (3.4%)	14 (2.6%)	0.574	1.331 (0.5–3.8)
Bacterial disease (unspecified)	n/a	n/a	n/a	n/a	7 (4.7%)	5 (0.9%)	0.005	5.368 (1.7–17.2)
**Primary diagnosis**								
Heart disease	34 (13.5%)	74 (8.1%)	0.008	1.777 (1.2–2.7)	5 (3.9%)	15 (3.1%)	0.781	1.240 (0.4–3.5)
Renal failure	19 (7.5%)	38 (4.1%)	0.027	1.886 (1.1–3.3)	7 (5.5%)	16 (3.4%)	0.300	1.646 (0.7–4.1)
Complications of surgical and medical care	17 (6.8%)	31 (34.0%)	0.017	2.068 (1.1–3.8)	n/a	n/a	n/a	n/a
Bacterial disease (unspecified)	14 (5.6%)	51 (5.6%)	0.977	0.999 (0.5–1.8)	10 (7.9%)	10 (2.1%)	0.001	3.878 (1.6–9.5)
**Location of participant before admission**								
**Home**	**134** **(53.0%)**	**605** ** (65.7%)**	**<0.001**	**0.588** ** (0.4–0.8)**	**102** ** (72.4%)**	**452** ** (84.8%)**	**0.001**	**0.532** ** (0.4–0.8)**
Emergency department	48 (19.0%)	106 (11.5%)	0.002	1.800 (1.2- 2.6)	10 (7.1%)	18 (3.4%)	0.057	2.125 (1.0–4.7)
Assisted living	3 (1.2%)	1 (0.1%)	0.033	11.040 1.1–106.6)	3 (2.1%)	7 (1.3%)	0.451	1.595 (0.4–6.2)
**Had a previous admission**	**129** ** (51.0%)**	**301** ** (32.7%)**	**<0.001**	**1.977** ** (1.5–2.6)**	**85** ** (53.8%)**	**193** ** (33.0%)**	**<0.001**	**2.154** ** (1.5–3.1)**
**Diagnostic tests**								
Tested for CDI before the INDEX sample	80 (31.6%)	3 (0.3%)	<0.001	220.345 (53.7–904.5)	34 (25.0%)	95 (19.1%)	0.131	1.411 (0.9–2.2)
Positive toxin test before the index sample	9^b^ (3.6%)	1^a^ (0.1%)	0.323	32.76 (4.13-259.82)	12^b^ (7.6%)	2^a^ (2.1%)	<0.001	38.37 (8.89-165.68)
Tested for CDI after the INDEX sample	160 (63.2%)	252 (27.4%)	<0.001	2.921 (2.1–4.0)	50 (40.7%)	88 (19.3%)	<0.001	2.856 (1.9–4.4)
Positive toxin test after the index sample	53 (20.9%)	1^a^ (0.1%)	<0.001	124.3 (17.0–910.7)	15 (30.0%)	4^a^ (4.5%)	<0.001	8.693 (2.6–29.2)
**Co-morbidities**								
Congestive Heart Failure	134 (53.0%)	342 (37.1%)	<0.001	1.906 (1.4–2.5)	22 (15.1%)	73 (13.9%)	0.703	1.106 (0.7–1.9)
Chronic kidney disease	91 (36.0%)	216 (23.5%)	<0.001	1.833 (1.4–2.5)	24 (16.4%)	60 (11.4%)	0.100	1.534 (0.9–2.6)
Peripheral Vascular Disease	58 (22.9%)	148 (15.5%)	0.011	1.554 (1.1–2.2)	27 (18.5%)	69 (13.1%)	0.097	1.509 (0.9–2.5)
Chemotherapy in the 12 weeks preceding the INDEX sample	47 (18.6%)	239 (26.0%)	0.016	0.651 (0.5–0.9)	4 (2.7%)	43 (8.2%)	0.026	0318 (0.1–0.9)
Myocardial Infarction	43 (17.0%)	95 (10.3%)	0.003	1.780 (1.2–2.6)	9 (6.2%)	23 (4.4%)	0.380	1.442 (0.7–3.2)
Renal replacement therapy (e.g. dialysis) within 7 days of index sample	42 (16.6%)	82 (8.9%)	<0.001	2.037 (1.4–3.0)	4 (2.7%)	15 (2.8%)	1.000	0.963 (0.3–2.9)
Diabetes with organ damage	31 (12.3%)	57 (6.2%)	0.001	2.117 (1.3–3.4)	7 (4.8%)	32 (6.1%)	0.717	0.781 (0.3–1.8)
Cerebrovascular disease	37 (14.6%)	143 (15.5%)	0.724	0.932 (0.6–1.4)	23 (15.8%)	44 (8.3%)	0.008	2.057 (1.2–3.5)
Urinary tract infection	30 (11.9%)	93 (10.1%)	0.418	1.198 (0.8–1.9)	21 (14.4%)	36 (6.8%)	0.004	2.296 (1.3–4.1)
Dementia	8 (3.2%)	31 (3.4%)	1.000	0.937 (0.4–2.1)	17 (11.6%)	26 (4.9%)	0.003	2.544 (1.3–4.8)
Chronic obstruction pulmonary disease	26 (10.3%)	88 (9.6%)	0.731	1.084 (0.7–1.7)	17 (11.6%)	32 (6.1%)	0.021	2.042 (1.1–3.8)
**Charlson Comorbidity Index**								
0	4 (1.6%)	65 (7.1%)	–	–	31 (21.2%)	163 (30.9%)	–	–
1	7 (2.8%)	41 (4.5%)	–	–	19 (13.0%)	52 (9.9%)	–	–
2	19 (7.5%)	110 (11.9%)	–	–	8 (8.1%)	83 (15.7%)	–	–
3	28 (11.1%)	105 (11.4%)	–	–	13 (8.9%)	59 (11.2%)	–	–
**≥3 vs**. **<3**	**223** ** (88.1%)**	**705** ** (76.5%)**	**<0.001**	**2.277** ** (1.5–3.4)**	**81** ** (55.5%)**	**229** ** (43.5%)**	**0.009**	**1.627** ** (1.1–2.4)**
**≥4 vs**. **<4**	**195** ** (77.1%)**	**600** ** (65.1%)**	**<0.001**	**1.799** ** (1.3–2.5)**	**68** ** (46.6%)**	**170** ** (32.3%)**	**0.001**	**1.836** ** (1.3- 2.7)**
**Jonckheere–Terpstra** ** (ascending ordinal comparison of Charlson Comorbidity index)**	**253** ** (100.0%)**	**921** ** (100.0%)**	**<0.001**	**n/a**	**158** ** (100)**	**584** ** (100)**	**0.0015**	**n/a**
**Antibiotics**								
**At least one antibiotic** **(any class)**	**240** ** (85.0%)**	**893** ** (74.7%)**	**<0.001**	**1.3** ** (1.1-1.4)**	**106** ** (67.1%)**	**264** ** (45.3%)**	**<0.001**	**1.3** ** (1.1-1.5)**
**Cephalosporins**	**19** ** (8.9%)**	**27** ** (2.9%)**	**0.001**	**2.689** ** (1.5–4.9)**	**28** ** (13.9%)**	**34** ** (6.8%)**	**0.003**	**2.210** ** (1.3–3.8)**
Third Generation Cephalosporins	46 (21.6%)	120 (13.0%)	0.037	1.483 (1.0–2.2)	24 (11.9%)	47 (9.4%)	0.320	1.302 (0.8–2.2)
**Other betalactams**	**15** ** (7.0%)**	**21** ** (2.3%)**	**0.003**	**2.701** ** (1.4–5.3)**	**9** ** (4.5%)**	**6** ** (1.2%)**	**0.016**	**3.847** ** (1.4–11.0)**
Glycopeptides	63 (39.6%)	86 (9.3%)	<0.001	3.219 (2.2–4.6)	10 (5.0%)	35 (7.0%)	0.318	0.693 (0.3–1.4)
Fluoroquinolones	55 (25.8%)	144 (15.6%)	0.022	1.499 (1.1–2.1)	35 (17.3%)	89 (17.2%)	0.959	1.011 (0.7–1.6)
Meropenem	45 (17.8%)	99 (10.7%)	0.003	1.796 (1.2–2.6)	n/a	n/a	n/a	n/a
Other antibiotics	32 (13.3%)	44 (4.9%)	<0.001	2.886 (1.8–4.7)	22 (10.9%)	63 (12.6%)	0.535	0.850 (0.5–1.4)
Sulphonamides	46 (19.2%)	147 (16.5)	0.399	ns	4 (2.0%)	40 (8.0%)	0.02	0.233 (0.1–0.7)
**Indication for antibiotics**								
Infection other than CDI	133 (31.3%)	224 (18.4%)	<0.001	2.019 (1.6–2.6)	83 (52.5)	133 (22.8)	<0.0001	3.752 (2.599–5.418)
Prophylaxis	63 (14.8%)	243 (20.0%)	0.019	0.698 (0.5–0.9)	5 (3.3%)	28 (9.0%)	0.033	0.349 (0.1–0.9)
Pneumonia	63 (14.8%)	251 (20.6%)	0.009	0.670 (0.5–0.9)	38 (25.2%)	48 (15.3%)	0.011	1.857 (1.1–3.0)
Abscess	1 (0.2%)	20 (1.6%)	0.023	0.141 (0.0–1.1)	n/a	n/a	n/a	n/a
Fever	68 (16.0%)	239 (19.6%)	0.098	0.779 (0.6–1.0)	5 (3.3%)	25 (11.2%)	0.004	0.272 (0.1–0.7)
Neutropenia	n/a	n/a	n/a	n/a	0 (0.0%)	15 (4.8%)	0.004	1.000
**Antibiotic route**								
IV	378 (74.0%)	1031 (74.3%)	0.873	0.981 (0.8–1.2)	97 (62.2%)	241 (73.3%)	0.013	0.600 (0.4–0.9)
Oral	127 (24.9%)	335 (24.2%)	0.752	1.039 (0.8–1.3)	57 (36.5%)	88 (26.8%)	0.028	1.577 (1.0–2.4)
**Other medications**								
PPIs given	192 (76.9%)	592 (64.3%)	0.001	1.749 (1.3–2.4)	61 (48.8%)	202 (41.4%)	0.135	1.349 (0.9–2.0)
**Indication for PPIs**								
Prophylaxis	194 (88.2%)	620 (96.6%)	<0.001	0.265 (0.1–4.8)	9 (52.9%)	39 (48.8%)	0.795	1.183 (0.4–3.4)
Disease of the esophagus, duodenum and stomach	17 (7.7%)	14 (2.2%)	<0.001	3.757 (1.8–7.8)	n/a	n/a	n/a	n/a
At least one GI drug given (other than PPIs)	162 (64.0%)	512 (55.6%)	0.013	1.438 (1.1–1.9)	36 (30.3%)	144 (32.3%)	0.672	0.910 (0.6–1.4)
**Indication for GI drugs**								
Constipation	108 (35.0%)	417 (43.0%)	0.012	0.713 (0.5–0.9)	1 (5.0%)	13 (18.3%)	0.290	0.235 (0.0–1.9)
Ulcer prophylaxis	7 (2.3%)	0 (0.0%)	<0.001	1.000	n/a	n/a	n/a	n/a
Abdominal pain	16 (5.2%)	6 (0.6%)	<0.001	8.774 (3.4–22.6)	n/a	n/a	n/a	n/a
Chemotherapy	13 (4.2%)	1 (0.1%)	<0.001	42.557 (5.5–326.7)	n/a	n/a	n/a	n/a
Prophylaxis	23 (7.4%)	101 (10.4%)	0.125	0.692 (0.4–1.1)	9 (50.0%)	13 (18.3%)	0.020	3.650 (1.3–10.6)
**Route of administration for GI drugs**								
IV	86 (24.3%)	249 (23.0%)	0.603	1.077 (0.8–1.4)	3 (9.4%)	26 (28.0%)	0.050	0.267 (0.1–1.0)
Oral	260 (73.5%)	800 (73.7%)	0.915	0.985(0.8–1.3)	29 (90.6%)	64 (68.8%)	0.018	4.380 (1.2–15.6)
**Surgery and GI interventions**								
**Had surgery in the preceding 12 weeks**	**104** ** (41.1%)**	**259** ** (28.1%)**	**<0.001**	**1.784** ** (1.3–2.4)**	**39** ** (26.4%)**	**82** ** (15.7%)**	**0.003**	**1.920** ** (1.2–3.0)**
Elective	82 (78.1%)	173 (67.1%)	0.037	1.752 (1.0–3.0)	20 (60.6%)	35 (56.5%)	0.696	1.187 (0.5–2.8)
Acute	23 (21.9%)	85 (33.0%)	0.037	0.571 (0.3–1.0)	13 (39.4%)	27 (43.6%)	0.696	0.843 (0.4–2.0)
**Surgery type**								
Vascular surgery	24 (22.9%)	28 (10.8%)	0.003	2.444 (1.3–4.5)	6 (14.0%)	11 (13.9%)	0.784	1.174 (0.4–3.4)
Had a GI intervention	39 (15.4%)	53 (5.8%)	<0.001	2.985 (1.9–4.6)	32 (22.5%)	79 (15.6%)	0.054	1.569 (1.0–2.5)
Nasogastric tube placement	2 (5.1%)	11 (20.8%)	0.038	0.206 (0.0–1.0)	6 (26.1%)	22 (30.1%)	0.345	0.587 (0.2–1.6)
Endoscopy	27 (69.2%)	24 (45.3%)	0.022	2.719 (1.1–6.5)	16 (69.6%)	39 (53.4%)	0.951	0.974 (0.4–2.2)

*na, data not available as either not collected or wasn't in the top ten for that cohort, so was not analyzed. Variables where there was no significance found between cases or controls in either cohort are not displayed*.

a *Indicates control samples with either a previous or subsequent positive test for CDI outside the 8 week window for exclusion in the Univariate analyses*.

b *Indicates cases removed from multivariate analyses, to avoid inclusion of potential recurrent cases*.

*ns, not significant*.

The reason for admission and the medical specialty ward where the subject was located at the time index sample was taken were examined. In the PSS group there were significantly more cases than controls located in renal medicine/nephrology and general surgery wards (all *P* < 0.05). In the EUCLID group, there were no ward locations significantly associated with cases; gastroenterology ward was however significantly associated with controls (*p* = 0.012; Table 2). At the PSS hospital, significantly more controls were admitted with malignant neoplasms than cases (*p* = 0.017), and renal failure was the only reason for admission that was significantly more frequent in cases than controls (*p* = 0.015). In contrast, EUCLID cases were significantly more likely to have been admitted with and have a primary diagnosis of “bacterial disease (unspecified)” than controls (*p* = 0.005 and 0.001, respectively, Table 2).

EUCLID cases were significantly more likely than controls to be tested for CDI again after the index sample (40.7 vs. 19.3%, respectively, *p* < 0.001; Table 2). The length of time between the index sample and a subsequent sample being taken for testing was 21 days longer in cases than controls (median 37 vs. 16 days, *p* = 0.005; Table 3). In addition, cases were also significantly more likely to have a positive test result for *C. difficile* toxin in samples taken both before and after the index sample (previous positive 7.6 vs. 2.1% cases and controls, respectively, *p* < 0.001; post-positive 30.0 vs. 4.5% cases and controls, respectively, *p* < 0.001; Table 2). Only 3.8 and 7.6% of cases likely represent recurrence (PSS and EUCLID, respectively). For the PSS group, cases were significantly more likely to be tested for CDI both before and after the index sample than controls, possibly indicative of repeated episodes of diarrhea. Interestingly, however, samples from PSS cases were only more likely to be positive after an index sample. The majority of cases from both cohorts likely represent primary infection (96.2 and 92.4% for PSS and EUCLID, respectively).

**Table 3:** Metric variables with a statistically significant difference between cases and controls within at least one participant cohort.

		**PSS**			**EUCLID**		
**Variable**	**Statistical test**	**Number of days or blood test value (cases)**	**Number of days or blood test value (controls)**	***P*-value**	**Number of days or blood test value (cases)**	**Number of days or blood test value (controls)**	**P value**
Days from admission to sample tested (all participants)	*T*-test (mean)	15	10	<0.001	10	9	0.384
	Mann–Whitney (median)	10	8	0.001	6	3	0.028
Days from admission to sample tested (Participants admitted from their own home)	*T*-test (mean)	15	11	<0.001	8	7	0.575
	Mann–Whitney (median)	10	8	0.028	5	2	0.016
Days from admission to sample tested (Participants admitted from another Hospital)	*T*-test (mean)	19	10	<0.001	22	13	0.225
	Mann–Whitney (median)	12	7	0.001	6	9	0.221
Days from admission to sample tested (Participants admitted from the Emergency department)	*T*-test (mean)	12	8	0.039	8	13	0.337
	Mann–Whitney (median)	8	5	0.368	9	10	0.562
Days before the Index sample when a CDI test occurred (this could occur before the admission of interest)	*T*-test (mean)	28.9	11.9	0.0003	57	30	0.002
	Mann–Whitney (median)	12.5	176	0.232	37	16	0.005
Duration of antibiotics	*T*-test (mean)	6.3	8.7	<0.001	12	13	0.900
	Mann–Whitney (median)	6	4	<0.001	7	7	0.541
Duration of PPIs	*T*-test (mean)	17	12	0.0003	104	131	0.786
	Mann–Whitney (median)	11	8	0.0001	17	11	0.688
Duration of GI drugs	*T*-test (mean)	8	5	<0.001	24	91	0.379
	Mann–Whitney (median)	3	3	0.0001	4	5	0.779
Duration of steroids	*T*-test (mean)	16	8	<0.001	12	60	0.546
	Mann–Whitney (median)	7	4	<0.001	12	8	0.717
Days from surgery to Index sample	*T*-test (mean)	17	12	0.002	26	19	0.090
	Mann–Whitney (median)	10	8	0.125	22	15	0.131
Serum creatinine (mg/dL)	*T*-test (mean)	2.3	1.7	0.0001	1.5	1.2	0.023
	Mann–Whitney (median)	1.3	1	<0.001	1.0	0.8	0.023
White cell count (mm^3^)	*T*-test (mean)	13.2	12.6	0.759	13.0	9.7	<0.001
	Mann–Whitney (median)	11.4	9.4	<0.001	11.6	8.5	<0.001
Serum albumin (g/L)	*T*-test (mean)	27.7	29.4	0.013	26	28.6	0.227
	Mann–Whitney (median)	27	29	0.014	27	31	0.110

Co-morbidities with higher prevalence in EUCLID cases than controls were: cerebrovascular disease, urinary tract infection (UTI), dementia, and chronic obstructive pulmonary disease (COPD; all *p* < 0.05; Table 2). Co-morbidities with higher prevalence in cases than controls in the PSS group were: congestive heart failure, chronic kidney disease, and renal replacement therapy, peripheral vascular disease, myocardial infarction, and diabetes with organ damage (all *p* < 0.05; Table 2). Cases in both groups were associated with higher Charlson comorbidity scores, with 55.5 vs. 43.5% (*p* = 0.009) in EUCLID patients with a score of >3, and 88.1 vs. 76.5% in the PSS group (*p* < 0.001; Table 2).

Antibiotics were more likely to have been prescribed for an infection (acute use) in cases of CDI, whereas in controls these were significantly associated with prophylactic use (Table 2). Use of at least one antibiotic of any class was significantly associated with cases in both groups (both *p* < 0.001). Broad-spectrum antibiotics commonly associated with CDI were significantly more likely to have been prescribed to cases than to controls (Table 2). The median duration of antibiotic treatment (days) was longer in cases than in controls in the PSS group only (6 vs. 4 days, *p* < 0.001; Table 3). Antibiotics given by the oral route were significantly more likely to be prescribed to cases than controls in the EUCLID group (36.5 vs. 26.8%; Table 2); no association with route of administration was noted in the PSS group.

There was significantly more PPI use in the cases than the controls (76.9 vs. 64.3%, *p* = 0.001) in the PSS group; no such difference was seen in the EUCLID group. Duration of PPI use (days) was longer in these PSS cases than in PSS controls (median 11 vs. 8 days, *p* = 0.001; Table 3). Drugs affecting the GI tract (other than PPIs) were also more commonly prescribed in cases than controls in both groups (PSS 64.0 vs. 55.6%, *p* = 0.013; EUCLID 45.0 vs. 18.3%, *p* = 0.02; Table 2). Chemotherapy was significantly more frequently seen in controls vs. cases (in both EUCLID and PSS groups) in the preceding 12 weeks (*p* = 0.026 and 0.016, respectively, Table 2).

Cases were significantly more likely to have had surgery in the preceding 12 weeks in both groups (PSS 41.1 vs. 28.1%, *p* < 0.001; EUCLID 26.4 vs. 15.7%, *p* = 0.003). In the PSS group, elective surgery was significantly more common in cases than controls (78.1 vs. 67.1%, respectively, *p* = 0.037; Table 2). In addition, these cases were also more likely to have had vascular surgery than controls (22.9 vs. 10.8%, *p* = 0.003; Table 2), and to have had a GI intervention (15.4 vs. 5.8%, *P* < 0.001; Table 2). There were no significant differences in the frequencies of particular types of surgery in the EUCLID group.

### Multivariate Analysis

After univariate analyses, risk factors that were significantly associated with the risk of CDI in both groups were age ≥65 years (ORs 1.84 PSS and 2.40 EUCLID), number of antibiotics (both ORs 1.3 per additional antibiotic), cephalosporin use (ORs 2.69 and 2.21, respectively), high Charlson co-morbidity score (ORs for score >3 2.28 and 1.63, respectively), surgery (ORs 1.78 and 1.92, respectively) and prior hospital admission (ORs 1.98 and 2.15, respectively; Table 2). In both groups antibiotics received by cases as treatment for infection (acute use) was associated with a significantly increased risk of CDI (ORs 2.02 and 3.75, respectively), whereas the prophylactic use of antibiotics in controls was associated with a decreased risk of CDI (ORs 0.70 and 0.35, respectively).

After removing all cases that had a previous toxin positive fecal sample (potential recurrent cases removed PSS = 9, EUCLID = 21), multivariate analysis was performed with data for 1,885 patients (PSS = 1,165, EUCLID 721). Age ≥ 65 years, the number of antibiotics received and prior hospital admission were the only risk factors identified in each group and overall (both groups combined) that were significantly associated with an increased risk of CDI (Table 4). Overall, the odds of developing CDI increased by 1.2 for each additional antibiotic prescribed (Table 4).

**Table 4:** Risk factors in common across groups following multivariate analysis.

	**PSS (*n* = 1165)**	**EUCLID (*n* = 721)**	**Combined (*n* = 1886)**
	OR (95% CI)	OR (95% CI)	OR (95% CI)
Age ≥ 65	1.6 (1.2–2.3)	2.5 (1.6–3.7)	1.9 (1.5–2.5)
Number of antibiotics	1.3 (1.1–1.4)	1.2 (1.1–1.4)	1.2 (1.1–1.3)
Prior hospital admission	1.9 (1.4–2.6)	1.6 (1.0–2.4)	1.7 (1.4–2.1)

*Controls with evidence of prior or subsequent CDI within 8 weeks of the index sample, and any cases with evidence of CDI positive within 8 weeks prior to the index sample were removed from multivariate analysis*.

## Discussion

In this comparison of risk factors for CDI between a single center and multicenter groups, the only risk factors that consistently remained significant after multivariate analysis were age ≥ 65 years, the number of antibiotics prescribed, and prior hospital admission. As would be expected with a disease where most cases are related to contact with a healthcare facility, prior hospital admission has been widely described as a risk factor for CDI (25, 26). Increased age and concomitant antibiotics are also frequently reported risk factors (15, 16, 18). For some authors, the impact of antibiotics on the gut microbiome makes them perhaps the most important risk factor (27). Indeed our study demonstrates that the risk of CDI increased by 1.2 for each additional antibiotic exposure (Table 4). Importantly, our results demonstrate these particular risk factors remained significant across different patient populations; vital information for those designing vaccine studies. Other CDI risk factors, however, were specific to only a single group, which emphasizes the effects of subject source on risk factor identification.

There were differences in the number of controls with malignant neoplasm between PSS and EUCLID, as the PSS hospital has a large cancer population. The difference in patient mix between the single study site (PSS) and the multiple site group (EUCLID) was also evident in the reason for admission. In the PSS group cases were significantly more likely to have been admitted with renal failure, whilst in the EUCLID group cases were significantly more likely to have been admitted with a “bacterial disease.” It could be hypothesized that patients with acute bacterial disease are more likely to receive antibiotics and therefore be at risk of CDI, indeed these patients had an odds ratio of 5.37, demonstrating this increased risk of becoming a case of CDI.

There were no co-morbidities identified as significant CDI risk factors that were in common across the two groups, although an increasing Charlson co-morbidity score was significantly associated (Table 2). This is reflective of the frail nature of older patients (who were significantly associated with CDI) and their likely increasingly complicated clinical picture. Several of the co-morbidities identified as risk factors, after univariate analyses, would likely drive increased antibiotic use; such as UTI, COPD, and diabetes (with organ damage). It is probable that diabetic patients have a higher number of admissions to hospitals than other patients, with leg/foot ulcers, thereby possibly driving a higher rate of antibiotic use. Indeed, co-morbidities previously associated with CDI have often included acute bacterial illnesses that would necessitate the use of antibiotics (25).

Unlike the PSS cases, the cases in the EUCLID group were not more likely to be prescribed broad spectrum agents, other than cephalosporins and beta-lactams. The use of large quantities of broad spectrum agents in the PSS group may again be reflective of the patient mix in this group; namely cancer patients. In addition, antibiotics in cases of CDI for both groups were more likely to have been prescribed for an infection (acute use), whereas antibiotics in controls were significantly associated with prophylactic use. We hypothesize that this is likely due to the shorter duration of antibiotics used for prophylaxis and/or because some antibiotics known to increase the risk for CDI, e.g., fluoroquinolones, are not as commonly used for prophylaxis. This aligns with the admission data, where cases were more likely to be admitted with acute bacterial illness.

In the PSS group, PPI use was associated with an increased risk for CDI (OR 1.8), however there were no significant findings associated with the use of PPIs in the EUCLID group. This may be a reflection of the smaller total *N* (sample size) in the EUCLID group, and the different patient mix as, in total, 66.8% of patients in the PSS group had a PPI, compared with just 42.9% of patients in the EUCLID group. In addition, duration of PPI use in the PSS cases was longer than the controls (median 11 vs. 8 days, *p* = 0.001). It should be noted however, that although there was no difference between PPI use in cases and controls in the EUCLID group, the overall duration of PPI use was longer than for the PSS group (Table 3). As described previously (19), the role of PPI use in CDI is controversial and may reflect the potential impact of ascertainment bias in the types of control patients in studies of this nature.

Cases in both groups were significantly more likely to have had surgery in the preceding 12 weeks by univariate analysis, in agreement with previously published data (25). There were no significant differences in the type of surgery for the EUCLID group however, whereas the PSS cases were associated with elective surgery, such as vascular surgery and GI intervention. Again there were a larger overall number of patients that had surgery in the PSS group (30.9%) than the EUCLID group (16.3%) and these differences probably reflect the patient mix. It is important to note however, that in our study surgery was no longer significant after multivariate analysis.

Our study does have some limitations; this was a retrospective case/control study, and for the EUCLID group was limited to a defined pre-set in-patient group (those that had been part of the original EUCLID study). This therefore made matching on age/gender very difficult, so matching for controls was geographical only (within the same hospital). It was important however to use this defined group as they had had their CDI diagnosed by a standard, recommended CDI testing algorithm (22–24). By using this defined group we removed any possible bias that may have been added by the inclusion of patients who were carriers of *C. difficile* but without true CDI (28), a potential limitation of other risk factors studies. We also ensured that the data was cleaned, to remove any possible recurrent cases, as the risk factors for these may be different.

Another limitation of using retrospective patient notes is that often the antibiotic data has been poorly recorded, and indeed we found that to be the case. This may have led to lack of power to associate risk of CDI with a particular class of antibiotic. Importantly, however, we have shown that the risk of CDI is significantly increased with each additional antibiotic added, regardless of class, with an odds ratio of 1.2. Indeed, given the lack of antibiotic data, this may actually be an underestimation of the true impact. In addition, the pattern of antibiotic use within an institution may have an effect on risk, however, by utilizing the cohort of 59 hospitals, this should have reduced any bias produced by one site. This again highlights the need to weigh single-center studies with caution when looking at risk factor data. The prevalence of CDI at a center may itself be a risk factor for further cases; the design of the study did not allow for us to determine a point prevalence rate for each center, although those for the EUCLID cohort are well described in the original paper (22). Once more, the multi-center cohort should limit the possible bias caused by different prevalence rates at difference centers. Finally, it should be noted that the collection of data from the EUCLID cohort was 2 years earlier than the PSS cohort, which may have influenced some of the risk factors.

This study highlights that age ≥65, increased use of antibiotics and prior hospital admission are identified as common risk factors for developing primary CDI across different background in-patient populations. This knowledge is vital to designing robust and feasible phase III vaccine studies.

## Data Availability Statement

The datasets generated for this study are available on request to the corresponding author.

## Ethics Statement

The studies involving human participants were reviewed and approved by Ethical approval for this study was gained from the University of Leeds (SoMREC/14/085) and in those participating countries where it was required (Germany, Hungary, Poland, Romania, and Slovakia). Ethical approval in Germany was sought on a regional (state) basis, in response to different regional requirements. Written informed consent from the participants' legal guardian/next of kin was not required to participate in this study in accordance with the national legislation and the institutional requirements.

## Author Contributions

KD is the guarantor of the article. The study was devised by KD, GD, MW, and LP. KD and GD were the European Coordinators. National Coordinators were IP, AK, IM, HP, EN, ON, BG and FB. OC and MV were Study Coordinators for the PSS group. The study analysis was performed by KD and CB. The manuscript draft was written by KD, with main edits by MW, JL, HY, BC, EG, and LP. All other authors reviewed the manuscript before submission. All authors approved this final version before submission.

## Conflict of Interest

KD has received research funding from Astellas Pharma Europe Ltd, Alere, bioMérieux, Cepheid, Pfizer, and Sanofi-Pasteur and has received honorarium from Astellas Pharma Europe Ltd., and Summit. MW has received research support from Abbott, Actelion, Alere, Astellas, Biomerieux, Cerexa, Cubist, Da Volterra, European Tissue Symposium, Merck, Sanofi-Pasteur, Summit, The Medicines Company and Qiagen, has consultancies and/or lecture honoraria in the past 2 years from Actelion, Alere, Astellas, Astra-Zeneca, Basilea, Bayer, Cubist, Durata, European Tissue Symposium, J&J, Merck, Nabriva, Novacta, Novartis, Optimer, Pfizer, Roche, Sanofi-Pasteur, and Seres and is on the speakers' bureau for Pfizer. JL, HY, BC, and EG are employed by Pfizer Inc. LP was employed by Pfizer Inc., at the time of study design, conduct, and preliminary analysis. OC is supported by the German Federal Ministry of Research and Education and the European Commission, and has received research grants from, is an advisor to, or received lecture honoraria from Actelion, Allecra Therapeutics, Amplyx, Astellas, Basilea, Biosys UK Limited, Cidara, Da Volterra, Entasis, F2G, Gilead, Grupo Biotoscana, Janssen Pharmaceuticals, Matinas, Medicines Company, MedPace, Melinta Therapeutics, Menarini Ricerche, Merck/MSD, Octapharma, Paratek Pharmaceuticals, Pfizer, PSI, Rempex, Scynexis, Seres Therapeutics, Tetraphase, Vical, outside the submitted work. MV is a consultant to: Berlin Chemie, MSD/Merck, and Astellas Pharma; has served at the speakers' bureau of: Astellas Pharma, Basilea, Gilead Sciences, Merck/MSD, Organobalance, and Pfizer; received research funding from: 3M, Astellas Pharma, DaVolterra, Gilead Sciences, Merck/MSD, Organobalance, and Seres Therapeutics. FB has received consultant fees from MSD. HP received an honorarium from Astellas Pharma Europe. The remaining authors declare that the research was conducted in the absence of any commercial or financial relationships that could be construed as a potential conflict of interest.
